# An Evaluation Process for IoT Platforms in Time-Sensitive Domains

**DOI:** 10.3390/s22239501

**Published:** 2022-12-05

**Authors:** Marisol García-Valls, Eva Palomar-Cosín

**Affiliations:** Universitat Politècnica de València, 46022 Valencia, Spain

**Keywords:** IoT platform evaluation, IoT evaluation process, time sensitive IoT platform, real time IoT, cyber physical systems platform, IoT web application, web front-end, IoT performance

## Abstract

Determining the temporal behavior of an IoT platform is of utmost importance as IoT systems are time-sensitive. IoT platforms play a central role in the operation of an IoT system, impacting the overall performance. As a result, initiating an IoT project without the exhaustive knowledge of such a core software piece may lead to a failed project if the finished systems do not meet the needed temporal response and scalability levels. Despite this fact, existing works on IoT software systems focus on the design and implementation of a particular system, providing a final evaluation as the validation. This is a risky approach as an incorrect decision on the core IoT platform may involve great monetary loss if the final evaluation proves that the system does not meet the expected validation criteria. To overcome this, we provide an evaluation process to determine the temporal behavior of IoT platforms to support early design decisions with respect to the appropriateness of the particular platform in its application as an IoT project. The process defines the steps towards the early evaluation of IoT platforms, ranging from the identification of the potential software items and the determination of the validation criteria to running the experiments and obtaining results. The process is exemplified on an exhaustive evaluation of a particular mainstream IoT platform for the case of a medical system for patient monitoring. In this time-sensitive scenario, results report the temporal behavior of the platform regarding the validation parameters expressed at the initial steps.

## 1. Introduction

As part of the CPS (cyber-physical systems) domain, IoT systems are immersed in a physical environment by means of the extensive deployment of sensors for monitoring and actuation over physical objects.

IoT technology has transformed in a manner similar to how most activities are undertaken in a number of domains. In the course of the last decade, IoT technologies progressively penetrated most application areas, spanning from agriculture [1,2,3,4], health [5,6,7], smart manufacturing and automation [8,9], or smart cities [10,11] to name a few. One of the major reasons is that IoT technology can transform the way in which many traditional human-intensive processes are performed, resulting in the automation of such activities and the decrease in human intervention.

Using large numbers of sensing and actuating nodes yields powerful infrastructures over which different applications can be developed that automate monitoring and actuation tasks on the targeted physical objects, such as the environment, patients, production machines, etc. Moreover, it supports adding logic that enhances the intelligence and capacities of current systems and their human users. For instance, IoT technologies applied to agriculture systems may yield significant savings in the usage of water for irrigation; applied to e-health, it yields real-time monitoring processes for patient conditions that can result in the earlier detection of health issues and informed decision making, among others.

As IoT is inherently linked to physical processes occuring within the physical environment, its operation often has *real-time requirements*. This means that sensing the environment and actuating upon it has to be performed within time bounds that are intrinsically associated with these physical processes. Then, outputs and responses need be produced before the process-dependant deadlines expire.

Given the need to deploy the IoT platforms in real scenarios, some of which are time-sensitive, the modules of such platforms and related software will have to prove compliance relative to given non-functional properties such as temporal behaviours or scalability capacities, among others. This should be performed before the final system’s design is performed and well before the actual implementation begins. Failing to perform this may lead to great monetary loss, e.g., the selected IoT platform proves to underperform once the final system experiences the testing phase.

The vision of a miriad of potential sensors being connected to a platform will have to find a realistic upper bound that proves to be sufficient and acceptable for the particular time-sensitive domain as indicated by the requirements obtained from domain experts. To design systems that meet non-functional properties such as time in IoT services, more knowledge about the behavior or the internal components is needed when handling time-sensitive interactions from IoT devices and nodes [12,13].

A number of contributions on defining software frameworks and platforms for IoT domains appeared in the last decade. Many of these contributions focus on the definition of programming interfaces for connecting IoT devices that can collect data sensed on the environmnet. Other contributions targeted the design and development of software libraries to provide collection and visualization capabilities for sensed data. That is, the majority of contributions target the functional side, data exchange, and visualization capacities. However, in IoT systems that have timing requirements, the overhead caused by the platform over communications must be assessed to determine if the achieved operation times are acceptable for the target system. This is essential when more and more software layers are added, e.g., web interfacing, that progressively increase the operation’s overhead.

Temporal evaluations are typically performed once the system is finalized as a means to validate the developed system. Waiving the initial and individual evaluation of key parts and technologies may yield a failed project. The key parts that require an initial evaluation are the core software layers, platforms, and external APIs that provide the fundamental logical infrastructure.

In this context, our work contributes to provide a mechanism for the early validation of a key software piece in IoT systems that is the central platform. In the rest of this paper, we refer to this central component as the IoT platform. The precise contributions of this paper are as follows:

- We define an evaluation process to assess the temporal behaviour of IoT platforms to determine their suitability for a given IoT project and application domain;
- We integrate non-functional properties such as scalability and stability in the proposed evaluation process.
- We analyse the internal components of IoT platforms and devices with respect to the different possible deployment architectures. As a result, we define different *architectural perspectives* and integrate them in the evaluation process. Moreover, we consider the execution infrastructure of the server side, which is a key pilar in the final deployment and impacts the temporal behavior.
- We validate and illustrate the proposed process on a particular mainstream IoT platform [14]; we perform an exhaustive experimental set for the validation and determination of the temporal behavior of the platform. By applying the proposed phases on this exhaustive evaluation, the temporal characteristics of the evaluated target are extracted. A discussion is provided that shows how the decision-making process benefits from these results.

This paper is structured as follows. Section 2 presents the state-of-the-art method with respect to contributions on the IoT system’s evaluation. Section 3 explains the considerations with respect to time-sensitive domains; moreover, it describes the proposed evaluation process Section 4.

## 2. Related Work

IoT technologies contribute to the development of smart systems that monitor and actuate real-time operations on the environment, leading to significant improvements, such as the rational usage of resources not only on the computational side but also on the nature and surrounding environment. This is a powerful motivation for successfully adopting IoT technology in the vast majority of application domains, including agriculture [1] and industrial systems [2]. One of the major categories of IoT system’s contributions is the design and development of sensors applied to particular domains, such as agriculture systems [15,16] for enabling smart and water-saving irrigation or applied to medical systems [17]. These are different from our approach in that they focus on the hardware design of IoT sensors.

Another major contribution is that of architecting system-level solutions for IoT-based systems that directly or indirectly impact resource efficiency and timely behavior. Some of these postpone the monitorization of errors to a post-development phase, such as [13].

Probably the third major category of works combinee various subdomains, such as wireless sensors, IoT smart devices, and the cloud for storing the monitored data, while also providing performance studies over the designed solution. The examples include [18,19], which undertake a performance analysis to extract the communication costs from wireless sensors relative to the cloud backend; other less mature works such as [20] use fog as a paradigm to increase performace. These works concentrate on the engineering process once the target system to be developed is decided. Moreover, they are silent about the particular characteristics of time-sensitive domains in what concerns real-time applications, including both hard and soft variants; these works do not focus on the original need of assessing the adequacy of the IoT platform that will be employed, which makes them quite different from what our contribution provides.

On the performance side, evaluations are mostly directed toward the networking protocols and traffic management. This is the case of [21], which explores the contributions from narrow band communications for IoT (NB-IoT) physical deployments. Other contributions from NB-IoT consider repetition characteristics (e.g., [22,23]) in performing link adaptations, which enable resource management with respect to improving energy savings, data rate, and coverage efficiency. On the communication middleware side, which is the software level, there are a number of contributions, such as [24], that explore the efficiency of different communication middleware software that implement different IoT protocols, such as AMQP, CoAP, or HTTP. All these works are different from ours in that they do not contemplate the existence of an IoT software platform that acts as the core for sensing and visualization functionalities provided for both the operator and physical objects that are monitored.

A more reduced number of contributions focus on particular IoT platform evaluations, such as [25], that do not stress the particular real-time properties of the target system; it provides a brief evaluation of an IoT software with no processes left behind and tailored fully to the IoT-framework platform. This framework uses P/S technology (RabbitMQ message bus) and a local scheme. Another similar approach is [26], which evaluates Fiware based solely on the cloud architectural model to identify the shortcomings of this software model. It is also an evaluation method that is fully tailored to a very particular model. The authors of [27] provide a generic evaluation model covering an extensive set of properties. However, it does not target particularly time-sensitive systems, nor does it apply the evaluation model to an exhaustive set of experiments with respect to performance. In summary, none of these works rely on the provisioning of an evaluation procedure for assessing the precise characteristics of time-sensitive applications, nor do they provide exhaustive evaluations other than alternative deployment architectures; additionally, they are not specialized in time-sensitive systems, deriving non-functional properties related to time such as stability and scalability and finding the limits of the operations of the platform.

## 3. Model

### 3.1. Time-Sensitive Systems

This section explains the different levels of time-sensitive applications and describes the acceptable response times in each of them, spanning from web applications to real-time systems.

Web front-ends of distributed systems have quite common temporal requirements across different domains [28]. Most statements here are largely agreed upon when referring to responsive web services. When referring to web services, 100 ms is excellent response time. Good response is still achieved if the time ranges between 100 ms to 200 ms. There is some room in between, which includes time ranges up to 1 s; however, it is quite common that response times over 1 s will lead to problems in the acceptability of the application. Others such as [29] mention that 10 s is the limit for retaining the attention of the user on the screen.

Real-time systems are the types of system where meeting execution deadlines is mandatory. They can be divided into hard and soft real-time systems [30]. Hard real-time systems have zero tolerance toward missing deadlines as these may lead to catastrophic consequences; in contrast, in soft real-time systems, some deadlines can be missed, leading to degraded performances.

IoT systems and CPS systems have, sometimes, real-time parts as they often appear in combination with process control systems (control systems), embedded systems, and wireless sensor networks.

Mixing web technology in a time-sensitive domain creates a particular mixture of properties. In fact, the complexity of current systems inevitably leads to a combination that offers powerful web-based front-ends and servers with high capacities for integrating functionality. This mixture has, currently, its greater exponent in IoT platforms that combine communication middleware technology and web techs to provide visualization power and large integration capacities.

Most IoT systems have time-sensitive operations; however, given the burst of the IoT domain, most contributions led to providing functional prototyping and left behind some traditional reliability processes such as evaluations.

In time-sensitive scenarios, communication times between servers running backend processing functions and the smart IoT devices are important validation parameters. A major outcome of a proposed IoT system is to provide an evaluation that determines the acceptability of the underlying software tower. For this purpose, obtaining a sufficient statistical representation from the experiments is necessary in order to determine whether the temporal behavior of the software’s logic running on the IoT infrastructure is valid.

**Figure 1:**
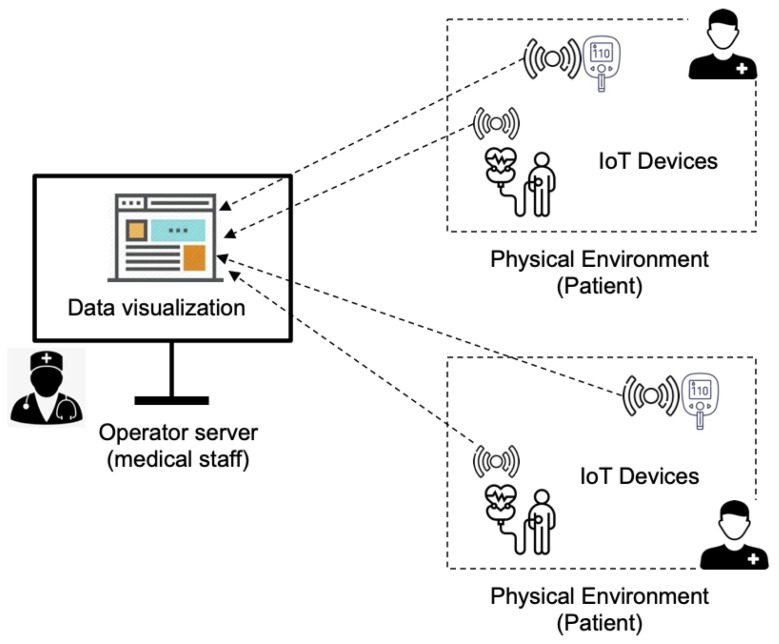
Overview of an IoT eHealth system for patient monitoring.

Figure 1 illustrates the target system that includes sensors for reporting the real-time readings of the vital signs of patients. Sensors are connected to a server by means of an IoT platform and a well-defined interface. The server receives the patient’s data in real-time; then, it processes the data that allow operators to detect alarms and/or reason about the particular health conditions. Security considerations in this scenario are left out of the contribution, although basic security assurance mechanisms are assumed, such as the findings in [31] for preventing well-known exploits on web interfaces.

In an IoT environment, communications between the platform and sensors are enabled by smart devices. At the resource-constrained level with low bandwiths, low battery, and reduced computation power, resource-efficient data exchange protocols are utilized. Various options exist from web-like client–server interactions such as [32] and publish–subscribe (P/S) protocols such as [33,34]. The constrained part of the IoT system typically communicates with the servers by means of a gateway (also smart IoT devices) using REST [35] communication schemes with protocols such as [36]. Additionally, real-time technology can be used in P/S schemes, such as [37]. This can be employed with a REST overlay protocol over the basic UDP scheme of DDS [37].

### 3.2. Evaluation Process

If inspiration from real-time systems is taken, time-sensitive domains employ system designs where the software side needs to be very efficient in its operation and in its resource consumption. This yields the construction of typically multi-threaded software that employs synchronization techniques at all levels, resulting in (although not always) higher speeds with respect to output generation. Analysing the internals of the platform and whether it employs multi-threading, performance APIs, etc., is an important aspect in this case. This will happen in the architectural perspective’s evaluation. Architectural perspectives can include the following: cloud, local, or intra-node (i.e., the analysis of the threading model employed, the programming employed, etc.).

**Figure 2:**
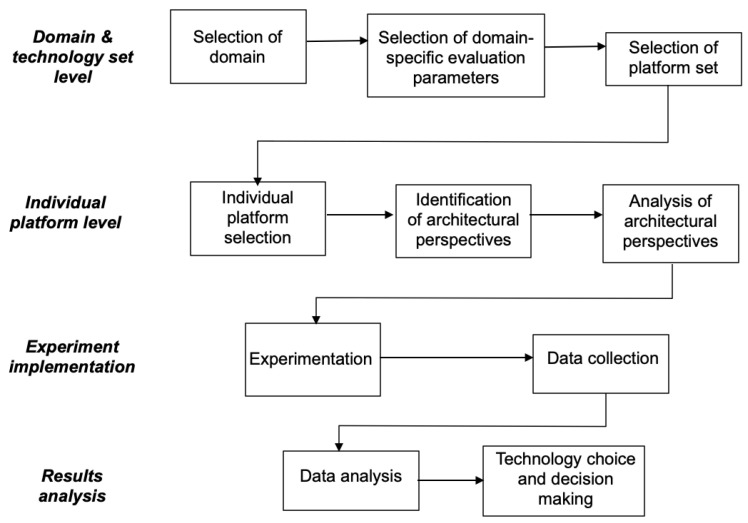
Evaluation process: phases and activities.

The phases are grouped into four levels: application domain and technology set, individual platform, experiments, and result analysis. Figure 2 shows the general scheme of the proposed evaluation process. Additionally, each phase has a number of activities that guide how the work is performed for that particular level.

***Phase 1—application and technology set***. This phase includes the activities related with the specification of the particular application domain in which the target system has to be developed and operated. Aspects related to the identification of the parameters of interest that will later be evaluated by the experiments are important here. The proper selection of the evaluation parameters will yield a proper selection of the technology universe that are candidates for the system’s development.

The activities comprised in this phase are as follows:

1. *Domain selection*. This activity collects information from similar domains and decides on the particular domain in order to select one that includes the refinement of the differentiating aspects and characteristics. For example, a medical system can be a type of real-time system; moreover, a real-time system can be either hard or soft. Temporal behavior matters in both subdomains, but differentiation is clue in terms of how the system is designed, developed, tested, and mantained. In this example, the result of this activity would be to select, e.g., the soft real-time domain as the one that best fits the system that will be developed.
2. *Domain-specific (general) evaluation-parameters selection*. After the domain selection, particular evaluation parameters are selected for that domain. This will yield a subset from the parameters shown in Table 1. Important parameters at the non-functional level are timeliness, resource efficiency (memory consumption, energy, etc.), usability (devices that can interface and be used in the system front-end), availability, and security (in the backend, all nodes, etc.) Moreover, together with the identification of the evaluation parameters, the acceptable values are set. We consider that functional parameters are evaluated after the system that is implemented.
3. *Technology set selection*. The outcomes of the first two activities (i.e., the precise domain selection and the extraction of the evaluation parameters) enables the determination of feasible candidate platforms that can be used to develop the target system. As an example, let us think of a domain that requires a high security level. In that context, a PHP backend will not be considered as part of the feasible technology universe for the targeted system.

***Phase 2—individual platform***. The second level concerns the study of the individual platforms and software pieces (incuding APIs, programs, software frameworks, platforms, and applications, among others) from the universe of technologies identified in phase 1. At the same time, each platform can be studied in different deployment scenarios. Moreover, different deployment scenarios may involve studying the platform from the point of view of its internal structure to derive the important characteristics about its behavior and even to explore its execution limits.

Activities in this phase include the following.

**Figure 3:**
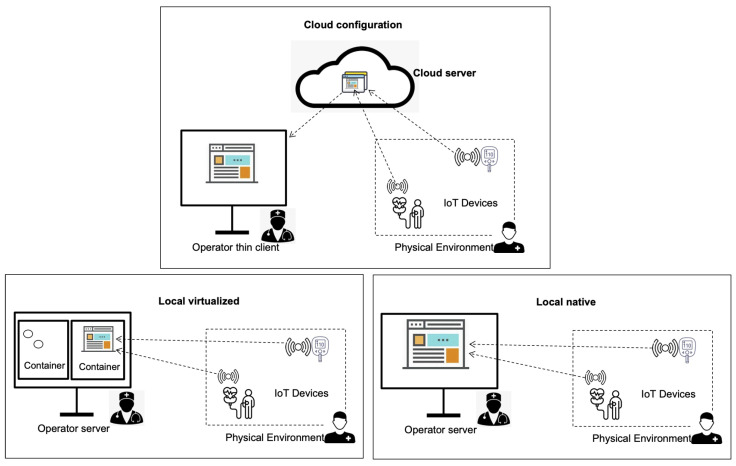
Architectural perspectives for IoT platform deployment.

1. *Individual platform evaluation*. This activity results in the analysis of the outcome of phase 1, i.e., the selection of the particular platform that preliminary is thought to be a suitable candidate for the development of the system. Then, this platform will be evaluated to explore its limits and to ensure that it provides the needed infrastructure.
2. *Architectural perspectives identification*. As the cloud-computing paradigm and container technology have become popular and are widely used, we frequently find that most platforms support different deployments. The current mainstream options are the following (see Figure 3):
  (a) Cloud (online);
  (b) Local-native;
  (c) Local-virtualized.
  In this paper, we refer to these deployment options as *architectural perspectives*. This activity deals with the identification of the actual architectural perspectives supported by the platform and the associated technologies that enable it.
3. *Analysis of platform architectural perspectives*. Prior to the system’s development, which deployment scenario will be employed is decided in order to analyse the full set of involved technologies. For example, if one of the architectural perspectives supported by the platform is *local-virtualized*, this activity will determine the type of container technologies employed in such a deployment, i.e., the third party technologies that are pulled-in by the platform that will also be evaluated.

***Phase 3—experiment design and implementation***. The activities related to how the experiments are designed and carried out are inluded in this phase. The experiments are designed to measure the target evaluation parameters selected in phase 1. The experiments are, then, conducted, and the results are collected.

The activities in this phase are the following:

1. *Experiment design*. This activity collects the results obtained from the “architectural perspective analysis”. In fact, the selected architectural perspective determines how the experiments are designed. Simultaneously, evaluation parameters are refined and selected. Lastly, during this activity, the experiments are run. It is of outmost importance that the experiments force the platform’s execution capabilities in order to explore its limits and to determine the points where the platform’s capabilities are exhausted.
2. *Data collection*. Data resulting from the experiments activity are collected and cleansed for analysis in the next phase.

***Phase 4—results analysis***. Activities dealing with the analysis of the results collected from the experiments are analysed in this phase.

The activities of this phase are as follows:

1. *Data analysis*. Data output from the experiments are analysed. Different analysis methods based on statistical measures, infered calculus, etc., can be employed. The experiments conclude which are the safe limits of the platform and confirm if the platform is valid (or not) for the particular application domain according to the evaluation parameters fixed in the early phases.
2. *Technology choice and decision making*. This activity comprises a discussion of the technology choice and suitability level of the particular technology in the application domain.

## 4. Design and Implementation

This section illustrates the evaluation process, including steps from phases 1 to 3; the experiments and validation (corresponding to phase 4) are provided in Section 4.2.

### 4.1. Phase 1—Domain and Technology Set

***Domain selection***. The target application domain includes medical systems for the remote monitoring of the health conditions of patients or elderly people. In the physical scenario, the monitored patients are located at individual rooms. The monitoring conditions are obtained from a number of sensors that gather patients’ ata and readings of environmental conditions. Patient signals/readings are displayed at a central panel and are operated by the server located at the nurse control point. The server is located within 100 m in distance from any monitoring sensor: an operator (nurse) is located within a maximum of 100 m from any monitored patient.

The set of potential evaluation parameters is identified at this phase. In this example, the parameters shown in Table 1 apply and they will be refined; a subset is selected in the subsequent activity.

***Domain-specific (general) evaluation-parameters selection***. This is a time-sensitive system as the readings of patients/elder with critical conditions should not undergo unbounded delays. Displaying data below the 10-s limit is acceptable. The reaction relative to detected abnormal conditions is possible and can result in proper treatments. This includes the communication costs from the smart sensor at the patient side to the server side’s processing operations and display at the operator (nurse) side. Derived from this scenario, the evaluation parameter is *data delivery-time*, and it comprises the temporal cost from the obtained signal until it is collected and displayed by the platform. Parameters of scalability and performance are also important in such a scenario.

Scalability is achieved if the platform supports request bursts of over 1000 requests without service degradation.

Performance is achieved if server response times never exceed 5 s.

***Technology set selection***. There are a number of platforms that can be part of the technology universe for this type of system. We focus on IoT platforms for IoT systems used in time-sensitive domains such as Industrial IoT and similar domains. They are employed in critical contexts such as manufacturing and production, and they shares common requirements with medical systems.

There are different possible deployments for the platform that correspond to the different architectural perspectives of the final system. These are Kaa [38], Losant [39], ThingsBoard [14], etc. These platforms are utilized in a number of IoT domains that require connections with multiple IoT devices and efficient resource utilization. All architectural perspectives are implemented. Other solutions such as [40,41,42] are intended for a core cloud server deployment.

In summary, the universe of suitable technologies conformed with [14,38,39].

### 4.2. Phase 2—Individual Platform

***Individual platform evaluation***. We select [14] given its target on industrial systems and IoT in general. Preliminar analysis (performed via a visual inspection and analysis of the tool’s technical documents [14]) reports good fault-tolerant capabilities, performance, and scalability; moreover, versatility, ease of use, and web capacities are high. Other factors such as its open source nature and community-active participants are also factors that have conformed to the decision.

***Architectural perspectives identification***. Since [14] supports all main architectural options, all deployment possibilities are also available for the target system: cloud, local-native, and local-virtualized.

***Analysis of architectural perspectives***. At this point, the different perspectives have to be analyzed:

- Cloud deployment. The platform is allocated in a distant remote server at some third-party hosting provider. In this scenario, the operator (nurse) server is closer to the patient (in the range of 100 m distance). Therefore, physical readings travel from patient to cloud and from cloud to operator server. The communication times in this scenario can be high, as the latency is aproximately twice the path from the patient to the actual cloud backend. Data have to be fed back from the cloud to the operator (nurse) server.
- Local-virtualized. The central platform is located in the operator server. This local platform is deployed on a container in the operator server, using some container technology, e.g., Docker. The physical readings are sent to the operator server (not to the cloud). The advantage of this approach is that virtualization supports higher scalability as new functionality and services can be added on the fly simply by an activation of new containers and services that have been previously downloaded.
- Local-native. In this perspective, the platform is directly hosted on the operating system of the operator server. As in the previous case, the patient’s readings are sent to the operator server (not to the cloud). In a native mode, communication times between patient IoT sensor and server are lower than in a local-virtualized environment. The reason is that there are no addition software layers (process level) located between the native (host OS and network driver) and the application platform. Nevertheless, it is also true that some technologies are optimized to achieve higher performances in virtualized environments. This information shows up in the experiment’s phase.

The output of this activity is the selection of the architectural perspectives that need be evaluated for the target software platform: (i) for cloud deployment and (ii) for local-virtualized. These are the perspectives best supported for the latest set of platform functions.

**Figure 4:**
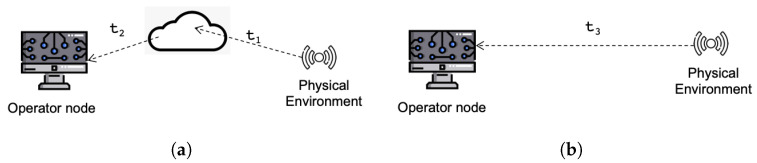
Communication times. (**a**) Cloud scheme. (**b**) Local scheme.

Table 2 shows the high level analysis of the architectural perspectives. The temporal measures refer to the scheme in Figure 4.

In Table 2, $t_{1}$ represents the communication cost between the sensing device and the cloud server; $t_{2}$ represents communication times between the cloud server and the operator server; both $t_{1}$ and $t_{2}$ refer to a cloud-only scheme. In a local scheme, there is no traffic relative to the Internet, so communication times are lower; in this case, the communication costs from the sensing device to the operator server is $t_{3}$ such that $t_{3}<t_{1}+t_{2}$.

$s_{c}$ and $s_{v}$ represent the scalability in a cloud-online and a local-virtualized sheme, respectively. $s_{c}$ and $s_{v}$ are empirically obtained values, derived from an actual evaluation of the target platform. Their values correspond to the size of the request bursts produced by requesting clients (sensor devices) when they are served, preserving the normal operation of the platform. The request burst size should be higher than the threshold set in phase 1 (see Table 3). It is common practice that an online deployment of an IoT platform supports fewer clients, whereas a local deployment can improve the number of supported clients and requests.

The stability measures are $l_{c}$ and $l_{v}$ for cloud and local, respectively. Stability is obtained at the point when the response time of the server is below that of an acceptable limit in all situations.

## 5. Validation and Results

This section describes phases 3 and 4, describing their constituent activities. For the sake of readability, we do not divide the subsequent subsections into their constituent activities.

*Experiments design* is triggered on the output of the previous phase. This involves architecting the particular experiments to be run on the platform for the selected architectural perspectives.

To evaluate service time or *latency*, the platform is instrumented to measure the communication costs between the client and server with multiple request bursts and data payloads.

To evaluate *scalability*, the platform undergoes several experiments that involve increasing the number of request bursts, as all IoT devices that may simultaneously need to send communication messages (data and state) to the platform. The platform must exhibit service times that remain around logical and reasonable values, adjusting the different request burst sizes.

For *data collection*, the platform is instrumented with a script code that collects data from all experiments. This is quite a large amount of traces that are presented in the graphs that follow. The data are explained to show the validity of the proposed evaluation process. 

### 5.1. Cloud Evaluation

Exhaustive sets of tests have been run that measure the latency and system operation as we vary the number of device invocations relative to the backend server side and the data payload.

The experimental setup is based on a full operational backend data center running the IoT platform based on a microservices architecture [41]. The IoT device’s cloud is emulated and it comprises low-power sensors that use an HTTP-enabled proximity gateway.

Sensor devices trigger communication messages relative to the server, generating a single data value on an increasing number of request bursts. They are emulated via the development of parallel sending messages. Increasing the payload is performed by increasing the number of accumulated data invocations. Sizes of the request’s burst range from low (10 requests), med (20 requests), and *med–high* (30 requests) to high (40 requests).

Experiments show two temporal costs: $cost_{1}$ and $cost_{2}$. $cost_{1}$ corresponds to the bare communication cost between devices and the server, i.e., the time elapsed from the instant when data are sent by the sensor and the time when it obtains the bare acknowledgement from the server. $cost_{2}$ corresponds to the full cost of the interaction, i.e., from the device request instant until the instant that the full response from the server reaches the IoT device. Time units are shown in miliseconds in all graphs.

**Figure 5:**
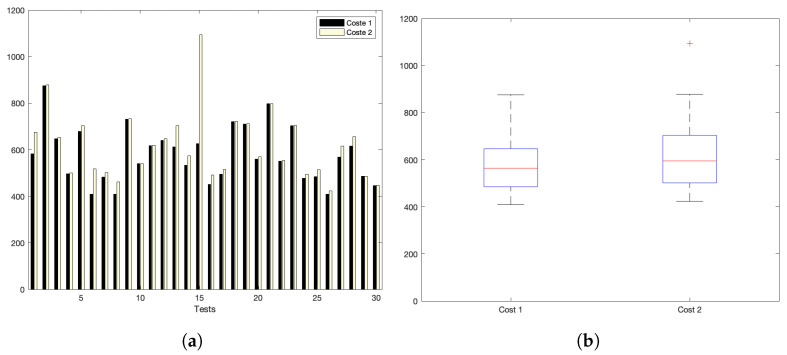
Low request rate: (**a**) individual test values for $cost_{1}$ and $cost_{2}$; (**b**) summary values.

**Figure 6:**
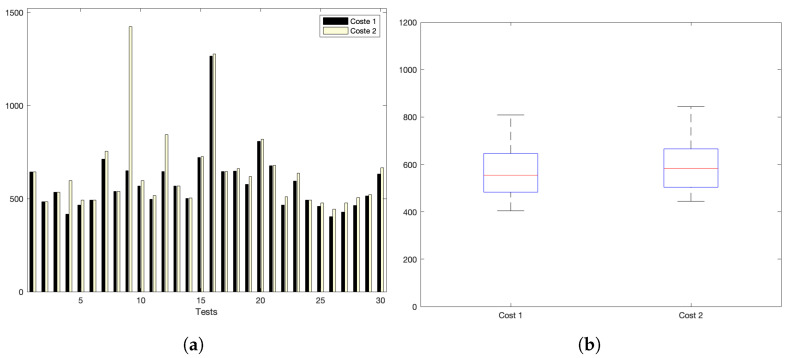
Med–low request-rate scenario: (**a**) individual test values for $cost_{1}$ and $cost_{2}$; (**b**) summary values.

**Figure 7:**
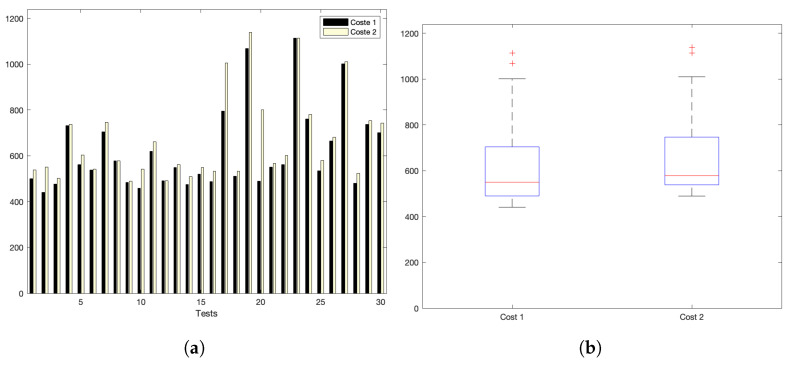
Medium request-rate scenario: (**a**) individual test values for $cost_{1}$ and $cost_{2}$; (**b**) summary values.

**Figure 8:**
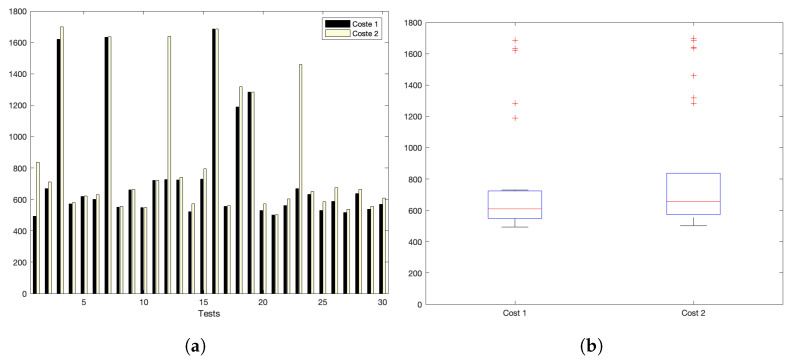
High request-rate scenario: (**a**) individual test values for $cost_{1}$ and $cost_{2}$; (**b**) summary values.

Figure 5a,b show the interaction costs for a scenario with little communication demands. The stress on the platform is progressively augmented, as shown in Figure 6a,b (for a medium–low request burst rate); Figure 7a,b (for a medium rate); and Figure 8a,b (for a high burst rate).

Part (a) of Figure 5, Figure 6, Figure 7 and Figure 8 show the actual costs of the server’s processing operations and overall communications. Parts (b) of Figure 5, Figure 6, Figure 7 and Figure 8 show the summary of costs in the form of the grouped percentiles of the occurrences of different temporal values.

As derived from these sets of exhaustive experiments, it is evidenced that the response time of the platform shows quite stable behaviours. Stability is derived from the fact that, as observed in the corresponding box plots, the 25% and 75% percentiles are consistently below 800 ms. Precisely, for the low and mid-low rates (shown in Figure 5 and Figure 6, respectively), this value falls to 700 ms. Outliers are at a minimum and consistently below 1200 ms for all scenarios: below 1200 ms for the medium request burst rate (see Figure 7a,b) and below 1800 ms for the highest stress situation (see Figure 8a,b).

**Figure 9:**
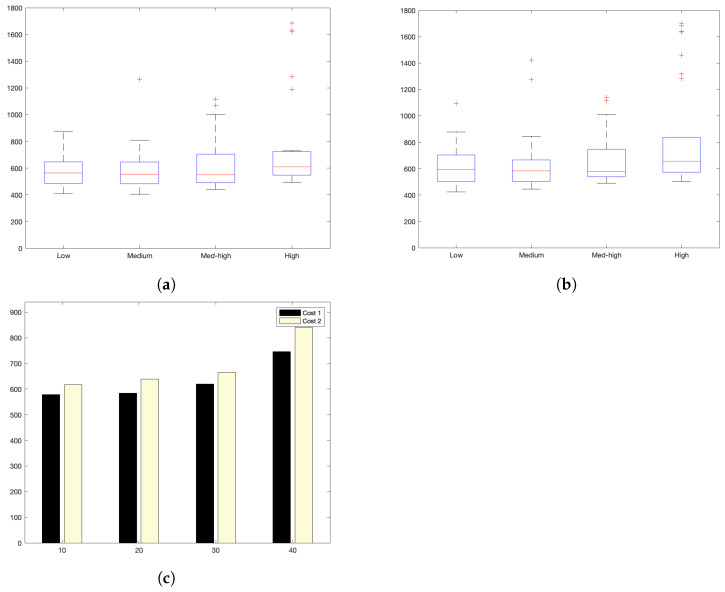
Summary of result analysis from all experiments on all scenarios from the cloud-online architectural perspective. (**a**) Summary of $cost_{1}$. (**b**) Summary of $cost_{2}$. (**c**) Average times for $cost_{1}$ and $cost_{2}$.

Additionally, Table 4 and Figure 9c reflect the stability of the platform, summarizing the average costs of each experiment.

The stability of the platform’s operation is also indicated by the calculated dispersion measure. For this, the standard deviation has been used (indicated in Table 5 and Table 6 as *Std. dev.*). Results show that the standard deviation is kept consistently around 34 ms; moreover, the load of the particular scenario does have an influence on this value.

Figure 9a,b graphically summarize the analysis of all results obtained from the experiments.

Then, the scalability for the cloud-online architectural perspective, that is, the number client devices that are properly handled by the platform, is $s_{v}=40$, which does not meet the needs set by the validation criteria: s>1000 (see Table 2). In a cloud deployment, this platform’s capabilities fall below what is needed in terms of scalability.

We conclude that the latency requirements and stability requirements are met for devices with set sizes of 40 or less. Higher demands with respect to the number of IoT devices should be handled with an alternative architectural deployment.

### 5.2. Virtualized Evaluation

Experiments designed for the virtualized architectural perspective are based on the same steps. That is, latency and system operation have been measured with the variation of the number of device invocations to the backend server-side and the data payload, therefore achieving scenarios with different load.

The platform has beeen instrumented in a similar way given that the installation of the software is local and on a virtualized container engine. This includes the installation of a local database for recording the system model and the interactions with devices: the full server functionality is now included locally.

In this set of experiments, the same checkpoints are used to extract values that lead to a truthful and directly comparible result across the different architectural perspectives. In this respect, devices communicate with the server passing a single data value on an increasing number of request bursts. Increasing the number of accumulated data invocations has the effect of increasing the data payload. Sizes of the request bursts range from low (10 requests); med (20 requests); *med–high* (30 requests); and high (40 requests). In this case, we also capture values for Cost1 and Cost1, having the same meaning as in the previous evaluation. Also, time units are given in miliseconds.

Figure 5a,b show the interaction costs for a scenario with little communication demands; The stress on the platform is progressively augmented as shown in Figure 6a,b (for a medium-low request burst rate); Figure 7a,b (for a medium rate); and Figure 8a,b (for a high burst rate).

In a virtualized architecture for [14], depending of the used data base, there are three types of images to install the IoT platform. These typically have associated a particular type of data base needs:

- Bare IoT platform. It is a single instance of the IoT platform with an integrated data base (HSQLDB [43] as a multi-threaded database for high performance operation). However, it is not recommended to evaluate or in production mode. It is suitable for development and automatic tests.
- Powered database. It refers to including some full fledged data base like PostgreSQL [44]. This is suitable for small server with medium memory capaciy that will be used with low-medium scenarios (i.e., few messages per second).
- Document-oriented data base. Typically a document-oriented data base with lower capacity and performance, lack of support for server-side scripting, and no support (or limited) for secondary indexing, among others. In web environments, it is typically the most recommended, but also requires to run on higher capacity servers (more memory).

The particularities of deciding what to use is based on the needs of the application. The powered data base option provides higher performance as the relational data base is programmed on a native C module.

After multiple tests, we found out that this option supports a significantly higher number of requests than that of the cloud version. Table 7 shows the results of an initial test that explored the limits of the platform determining that the maximum number of requests that would be fully received at the server is 3000. This has allowed to decide in a fully informed manner that we perform the subsequent tests within the safe limits of the platform, with a maximum of 2000 requests in use.

Similarly to the cloud evaluation, sensing devices perform the sending of a single data value on an increasing number of request bursts. Increasing the number of accumulated data invocations yields to higher payloads. Derived from the previous tests to find the safe utilization limit of the platfor, we now divide into the following scenarios for different request burst-rates: low (10 request bursts, medium, high. Sizes of the request burst range from low (10 requests); *low–med* (100 requests); med (1000 requests); and high (2000 requests).

**Figure 10:**
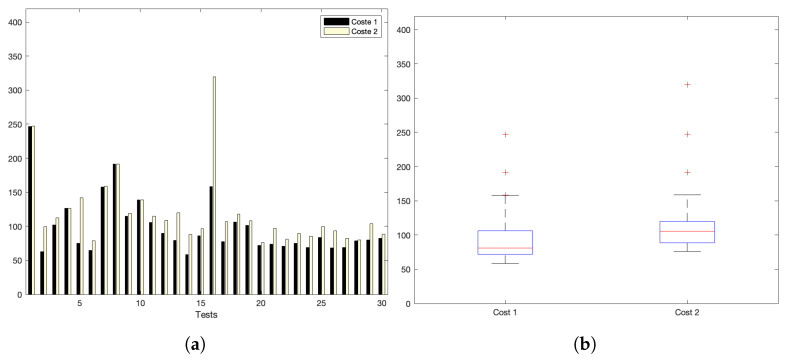
Low request burst-rate. (**a**) Individual test values for $cost_{1}$ and $cost_{2}$. (**b**) Summary of costs.

**Figure 11:**
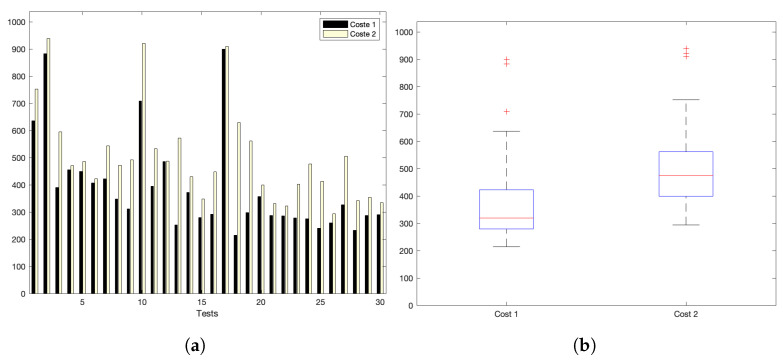
Med-low request burst-rate. (**a**) Individual test values for $cost_{1}$ and $cost_{2}$. (**b**) Summary values.

**Figure 12:**
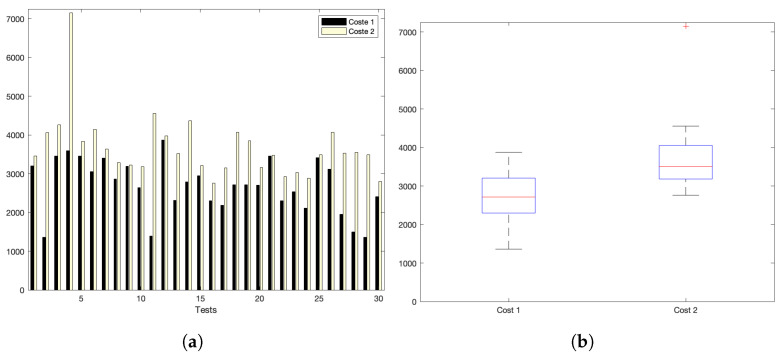
Medium request-burst rate. (**a**) Individual test values for $cost_{1}$ and $cost_{2}$. (**b**) Summary values.

**Figure 13:**
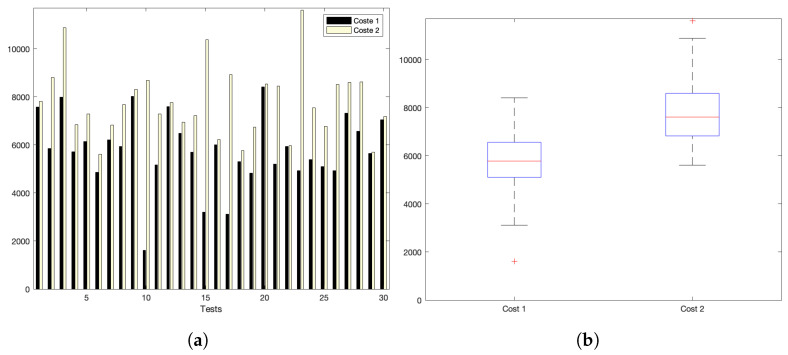
High request-burst rate. (**a**) Individual test values for $cost_{1}$ and $cost_{2}$. (**b**) Summary values.

Figure 10a shows the interaction costs for a scenario with low communication demands. The stress on the platform is progressively augmented as shown in Figure 11a for a medlow request burst rate; Figure 12a for a medium request burst rate; and Figure 13a for a high rate.

**Figure 14:**
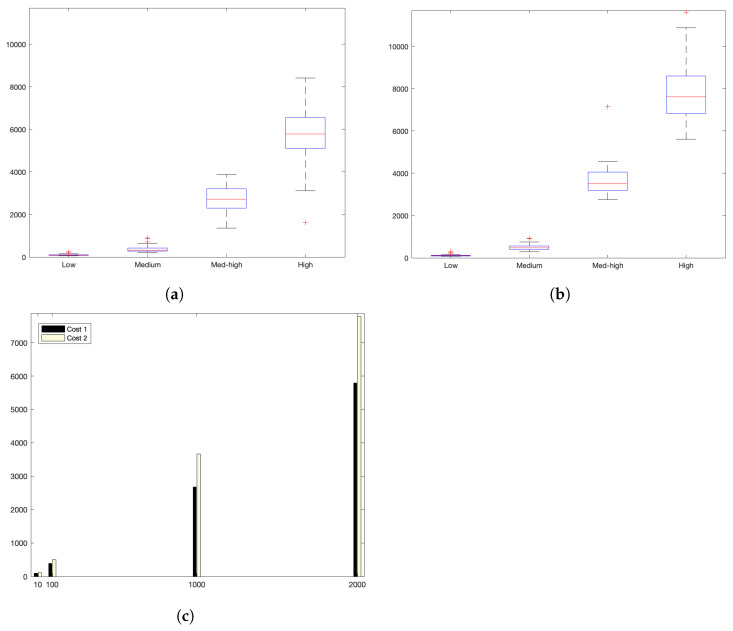
Summary of cost values for all scenarios. (**a**) $cost_{1}$. (**b**) $cost_{2}$. (**c**) Average costs.

Additionally, Table 8 and Figure 14 reflects the stability of the platform summarizing the average costs of each experiment.

Figure 14a,b summarize graphically the analysis of all results obtained from the experiments.

Analysing the previous experiments, it can be seen that the platform reflects a stable behaviour. Stability is derived from the fact that, as observed in the corresponding box plots, the 25% and 75% percentiles are below 600 ms for the low and medium rates. Precisely, for the low rate, this value falls down to 140 ms. Outliers are minimum and consistently below 350 ms and 920 ms for both scenarios, respectively; and below 4200 ms for the highest stress situation. Additionally, Table 8 and Figure 14 reflect the stability of the platform, summarizing the average costs of each experiment. The stability of the platform operation is also indicated by the calculated dispersion measure, precisely the standard deviation, that is coherent in its increase for scenarios with higher overload. It ranges from 13 ms for the low burst-rate scenario that is 16.25% of the average latency; 8.34% in the med-low; 2.41% for medium load situations; and 1.42% for the highest scenario. This evidences that the platform implements efficient data handling and communication mechanisms that are evidenced more significantly as for higher number of device interactions.

As a result, scalability in this particular architectural perspective is well supported as the number of client devices that are properly handled by the platform meets the validation criteria: $s_{v}=3000$ that is the size of the request burst that is analyzed exhibiting fully normal operation and efficient service times (see Table 7). Since the platform has been tested under a safe limit of $s_{v}=2000$ that is largely above the user requirements for the target medical domain, the platform is can be properly used in the implementation of the final system.

## 6. Conclusions

The large amount of contributions on the IoT literature focus on a preminent method for designing a target system with a validation of the particular designed solution. This paper has presented a different approach required in time-sensitive domains that need to have a premilinar guarantee on the appropriate temporal behavior of the core building block, such as the IoT platform. In this respect, this paper contributed an evaluation process for the IoT platform component of a time-sensitive system. We illustrated this evaluation on a medical systems scenario for which the evaluation parameters have been obtained a priori. The practicality of this approach lies in the fact that the IoT platform needs to provide sufficient guarantees for a scenario. It does not need to make comparisons with all existing possible variations and architectural views. Therefore, the paper has applied the process to the evaluation of a cloud deployment of an IoT platform for a medical system. We conducted exhaustive experiments for different load scenarios. We concluded that the experiments validate the use of this particular selected software for the described target medical system. All parameters show that latency, scalability, and stability are significantly below the requirements extracted from the application-domain experts.

## Figures and Tables

**Table 1:** Potential evaluation parameters—some examples. Some parameters can be expanded into a subset, e.g., *r* into *c* (processor time), etc.

Parameter	Description
Latency (*t*)	Communication time between sensing devices (clients) and server
Scalability (*s*)	Capacity to increase number of supported devices (i.e., large request bursts)
Stability (*l*)	Capacity to preserve normal operations and service in the event of increased data payloads
Availability (*a*)	Fraction of time when the system is up
Maintainability (*m*)	Capacity to update functionality over time
Resource usage (*r*)	Efficient usage of the computational resources such as the processor cycles, memory, bandwidth, etc.

**Table 2:** Selected architectural perspectives.

Architecture	Latency	Scalability	Stability
Cloud-online	$t=t_{1}+t_{2}$	$s_{c}$	$l_{c}$
Local-virtualized	$t=t_{3}$ where $t_{3}<t_{1}+t_{2}$	$s_{v}$ where $s_{v}>s_{c}$	$l_{v}$

**Table 3:** Selected evaluation parameters.

Eval. Parameter	Validation Criteria
Latency (*t*)	Valid if t<10 s
Scalability (*s*)	Valid if s>1000, where *s* is the size of the request bursts supported by the system, exibiting normal and full operation
Stability (*l*)	Validated via an analysis of standard deviation for different scenarios

**Table 4:** Latency summary for all scenarios (in miliseconds).

Test	$cost_{1}$	$cost_{2}$
**Low**	578.7589	616.9831
**Med–Low**	584.0259	638.4380
**Medium**	619.6905	665.5729
**High**	745.6596	840.4089

**Table 5:** Dispersion measures for $cost_{1}$.

	*Low*	*Med-Low*	*Med*	*High*
**Maximum**	875.826	808.639	1001.39	729.87
**Minimum**	409.451	404.284	440.91	493.360
**Average**	563.750	553.904	549.928	609.823
**Std. dev.**		35.5746	32.6910	34.4707

**Table 6:** Dispersion measures for $cost_{2}$.

	*Low*	*Med-Low*	*Med*	*High*
**Maximum**	877.832	843.822	1010.15	837.349
**Minimum**	423.348	444.357	489.666	502.309
**Average**	594.939	582.739	578.791	656.033
**Std. dev.**	33.0674	35.7347	33.3853	35.8154

**Table 7:** Relation between sent and received messages on a local-virtualized platform.

Request No.	Received Requests
50	50
100	100
500	500
750	750
1000	1000
1000	1000
2000	2000
2500	2500
3000	3000
3500	3228
4000	3763
5000	4910
6000	5845
10,000	9547

**Table 8:** Dispersion measures of latencies for all scenarios (in ms).

	*Low*		*Low-Med*		*Med*		*High*	
	${\mathrm{cost}}_{1}$	${\mathrm{cost}}_{2}$	${\mathrm{cost}}_{1}$	${\mathrm{cost}}_{2}$	${\mathrm{cost}}_{1}$	${\mathrm{cost}}_{2}$	${\mathrm{cost}}_{1}$	${\mathrm{cost}}_{2}$
**Maximum**	157.815	158.755	636.355	752.099	3872.19	4554.98	8408.11	10878.6
**Minimum**	58.4462	75.9995	214.225	293.345	1361.16	2759.53	3114.42	5608.42
**Average**	80.9318	105.273	319.437	474.983	2713.75	3507.42	5780.91	7609.75
**Std. dev.**	13.8380	13.8472	26.6307	30.15978		84.5573	40.1806	107.7098

## Data Availability

Not applicable.
